# Dietary Patterns and Practices Among Patients With Inflammatory Bowel Disease and Their Associations With Gastrointestinal Symptoms and Disease Activity: A Systematic Review

**DOI:** 10.7759/cureus.117475

**Published:** 2026-10-05

**Authors:** Adel F Alzahrani, Mohammad s Numan, Abdulrahman H Algrigri, Abdullaziz M Dwaib, Yazed M Alayesh, Mousa F Allebdi, Tariq F Alsubhi, Wed W Baabdullah

**Affiliations:** 1 Department of Gastroenterology, Endoscopy Unit, King Fahad Armed Forces Hospital, Jeddah, SAU; 2 Department of Gastroenterology, Endoscopy Unit, Ministry of Health, Jeddah, SAU

**Keywords:** dietary patterns, disease activity, food avoidance, gastrointestinal symptoms, inflammatory bowel disease

## Abstract

Patients with inflammatory bowel disease (IBD) often report food avoidance, restrictive eating, and dietary modification, although the association of these dietary behaviours with gastrointestinal symptoms and objective measures of inflammatory activity remains unclear. Clinically, it is important to distinguish dietary behaviours for symptom relief from dietary exposures associated with underlying inflammation.

The aim of this systematic review was to synthesise evidence on habitual dietary patterns and patient-adopted dietary practices among patients with Crohn’s disease, ulcerative colitis, or IBD-unclassified and to evaluate their associations with gastrointestinal symptoms and clinical, biochemical, endoscopic, or histological disease activity.

PubMed/MEDLINE, Scopus, and Web of Science Core Collection were searched for English-language observational studies published between January 1, 2016, and August 4, 2026. Eligible studies provided information on habitual dietary intake or diets used by patients in their daily practice or real-world situations. Prescribed therapeutic dietary interventions, supplementation studies, reviews, case reports, animal studies, and studies without IBD-specific outcome measures were excluded. The review was performed in accordance with Preferred Reporting Items for Systematic Reviews and Meta-Analyses (PRISMA) 2020, and methodological quality was evaluated using the Joanna Briggs Institute checklists for each design. Findings were synthesised narratively.

Eleven studies were included in this review, with 2,242 participants who had IBD. Across disease states, food avoidance, dietary restriction, meal skipping, and symptom-driven dietary modification were common. Three studies reported positive screening rates for avoidant/restrictive food intake disorder (10.2% to 32.5%). Dietary practices were restrictive and related to malnutrition risk and nutritional inadequacy, and low appetite and reduced food-related quality of life were also reported. Associations with dietary behaviours were more consistent for gastrointestinal symptoms than for objective measures of inflammatory activity, including C-reactive protein, faecal calprotectin, endoscopic activity, and validated disease-activity indices. No single dietary pattern or dietary total score was consistently associated with inflammatory activity.

Dietary patterns and practices in IBD were more consistently associated with gastrointestinal symptom burden than with objective measures of inflammatory activity. Self-imposed dietary restrictions may be associated with nutritional and psychosocial consequences. Patients with substantial dietary restriction should undergo assessment of inflammatory activity and nutritional status, with referral to an IBD-experienced dietitian when appropriate. Future studies using validated dietary instruments and standardised clinical, biochemical, endoscopic, and patient-reported outcomes are needed to clarify temporal and causal relationships.

## Introduction and background

Inflammatory bowel disease (IBD) is a group of chronic, immune-mediated inflammatory disorders of the gastrointestinal tract, primarily including Crohn’s disease (CD) and ulcerative colitis (UC). Both CD and UC can present with abdominal pain, constipation, bloody stool, urgency to defecate, and decreased quality of life; however, they differ in anatomical distribution, depth of inflammation, and clinical course [1]. Historically, the highest incidence of IBD has been in North America, Europe, and Oceania, but the incidence is rising in newly industrialised areas that are rapidly urbanising and making dietary changes [2]. An increasing burden has also been reported across the Arab world, including Saudi Arabia [3].

The aetiology of IBD is complex and multifactorial, including genetic susceptibility, immune dysregulation, changes in the gut microbiome, and environmental exposures [4]. This interest in diet is partly due to the fact that it is modifiable and can affect intestinal microbial composition, intestinal barrier integrity, mucosal immune responses, and the production of microbial metabolites. It is also a very important concern for patients who are attempting to determine which foods worsen symptoms or alter the disease course. However, the evidence for universal recommendations based on food to decrease the risk of relapse is limited. The International Organization for the Study of Inflammatory Bowel Diseases (IOIBD) has provided recommendations on various food groups and dietary factors, but with varied evidence for different exposures and different evidence for CD compared to UC [5].

Dietary patterns and dietary practices are different but related concepts. Dietary patterns describe the combinations, amounts, and frequencies of foods consumed regularly and may include Mediterranean, Western, plant-based, high-fat, high-sugar, high-fibre, low-fibre, and ultra-processed dietary patterns [6]. Dietary practices refer to actions taken in relation to disease or perceived symptoms, such as avoidance of certain foods, limiting certain food groups, reducing portions, skipping meals, fasting, reducing food during flares, or self-directed elimination foods. A systematic scoping review found food avoidance in 28-89% and restrictive dietary behaviours in 41-93% of adults with IBD [7].

Patients with IBD commonly perceive a relationship between food intake and gastrointestinal symptoms. A study of diet in IBD found that the majority of patients feel that diet plays a role in the initiation and management of their illness, and patients often change their diets in an effort to control their symptoms [8]. The direction of these relationships, however, is unclear. More severe symptoms may lead patients to avoid certain foods; therefore, observed associations between dietary restriction and symptoms may reflect reverse causality rather than an adverse effect of dietary restriction itself. The implication of reverse causality is especially pertinent in cross-sectional studies. Dietary habits also may differ by disease status, such that the more active the disease, the more severely restricted the dietary habits will be [7].

Long-term or excessive restriction can have nutritional effects. In one observational study, the prevalence of malnutrition was significantly greater and the consumption of calcium, vitamin A, and zinc was significantly lower in patients with IBD who excluded foods from their diet compared to those who did not [9,10]. Decreased food diversity can also lead to low energy and protein intake, micronutrient deficiencies, weight loss, and malnutrition [11]. Restrictive eating can also negatively affect food-related quality of life and social functioning. It remains challenging to differentiate restrictive eating from adaptive responses to gastrointestinal symptoms, and appropriate recognition and management are important in patients with IBD [9].

A central methodological issue is that gastrointestinal symptoms and inflammatory disease activity are related but not interchangeable outcomes. However, clinical and endoscopic remission may not be sufficient to ensure the resolution of symptoms due to functional or inflammatory mechanisms in the gastrointestinal tract. Patients with inactive IBD and ongoing gastrointestinal symptoms may also have a lower quality of dietary intake and food-related quality of life compared to patients without ongoing gastrointestinal symptoms [12]. Conversely, subclinical inflammation may occur despite limited symptom severity. Thus, treat-to-target guidelines differ from normalisation of inflammatory markers and endoscopic healing [13]. Associations between diet and validated disease-activity indices, C-reactive protein, faecal calprotectin, endoscopic activity, or histological inflammation should be analysed in isolation from diet-symptom associations.

Current literature has looked at therapeutic diets, food triggers, dietary beliefs, nutritional inadequacy, food avoidance, restrictive diets, and food-related quality of life. The choices of therapeutic diets and the behaviours of patients are, however, two different exposures in the methodological sense. Consistent terminology, use of self-reported questionnaires, validated dietary assessment instruments, and an accurate assessment of objective inflammatory activity were also noted in previous reviews [7]. However, it remains unclear whether patient-adopted dietary patterns and practices are more consistently associated with gastrointestinal symptoms, objective inflammatory activity, or both.

This systematic review aims to synthesise the evidence regarding habitual dietary patterns and patient-adopted dietary practices in IBD and their associations with gastrointestinal symptoms and disease activity. Associations between dietary patterns or practices and patient-reported gastrointestinal symptoms were evaluated separately from associations with clinical, biochemical, endoscopic, or histological measures of inflammatory activity. Food avoidance and dietary restriction are also summarised, and nutritional and psychosocial consequences are viewed as secondary outcomes. Prescribed therapeutic dietary interventions are excluded from studies that are primarily focused on this area.

## Review

Methods

Review Design and Reporting Framework

This systematic review was conducted to identify, critically assess, and synthesise all quantitative observational studies relating dietary patterns and dietary practices in people with inflammatory bowel disease (IBD) to gastrointestinal symptoms or disease activity. The review was conducted using a review question and a set of eligibility criteria, and the Preferred Reporting Items for Systematic Reviews and Meta-Analyses (PRISMA) 2020 Statement [14] was used for reporting. The review process involved a transparent and reproducible literature search, systematic study selection, data extraction, critical appraisal of methodological quality, and structured synthesis of the included evidence.

Review Question and PECO Framework

The review addressed the following question: What dietary patterns and dietary practices are reported among patients with inflammatory bowel disease, and how are these exposures associated with gastrointestinal symptoms and disease activity?

The question was framed using the Population-Exposure-Comparator-Outcome (PECO) format (Table 1) for consistency across the review objective, eligibility criteria, and evidence synthesis.

**Table 1:** PECO Framework

PECO	Description
P: Population	Crohn's disease, ulcerative colitis and inflammatory bowel disease (unclassified); patients diagnosed with these diseases.
E: Exposure	Habitual dietary patterns and patient-adopted dietary patterns, e.g., food avoidance and dietary restrictions, meal-related behaviours, fasting, reduced intake during disease flares, self-directed elimination, etc.
C: Comparator	Any patients with any different level (or category) of dietary exposure, those who do not adhere to the dietary practice in question, or another comparator as established by the original study. A comparator was not required for descriptive studies.
O: Outcomes	Patient-reported gastrointestinal symptoms, disease activity measures (clinical, biochemical, endoscopic, and histological).

Eligibility Criteria

Eligibility criteria were determined prior to the review in accordance with the objective of the review and the PECO framework.

Inclusion Criteria

Population: Patients with Crohn's disease, ulcerative colitis, and inflammatory bowel disease-unclassified (IBD-U), with a confirmed diagnosis according to clinical, endoscopic, radiological, histological, or physician-confirmed criteria. Studies of adult, paediatric, adolescent, or mixed-age populations were included. For studies involving patients with multiple gastrointestinal disorders, only studies that provided data separately for participants with IBD were included.

Exposure: Routine or real-world dietary patterns or practices that were part of a patient's normal lifestyle. A priori defined and/or empirically determined dietary patterns, dietary quality and dietary diversity, reduced dietary variety, fasting, food restriction, food avoidance, self-directed elimination practices, meal skipping, avoidance of perceived trigger foods, and dietary reduction during disease flares were all included among exposures eligible for inclusion.

Outcomes: Patient-reported gastrointestinal symptoms included abdominal pain, diarrhoea, bloating, abdominal distention, stool frequency, stool urgency, rectal bleeding, nausea, changes in stool consistency, and/or gastrointestinal symptom scores. Clinical outcomes included validated disease activity indices, physician assessment of disease activity, inflammatory markers, endoscopic/histological disease activity, relapses, hospitalisation, corticosteroid use, treatment escalation, and surgery due to IBD. Studies were included if they reported on the prevalence of dietary patterns or dietary practices.

Study design: Analytical cross-sectional studies, prospective or retrospective cohort studies, case-control studies, and other longitudinal observational studies were included. Baseline data from intervention studies were incorporated when reported separately from intervention results.

Publication characteristics: Full-text articles in English published in peer-reviewed journals from January 1, 2016, to August 4, 2026, were included.

Exclusion Criteria

Studies of exclusive or partial enteral nutrition (EN), parenteral nutrition (PN), Crohn's disease exclusion diets, specific carbohydrate diets, low-FODMAP (fermentable oligosaccharides, disaccharides, monosaccharides, and polyols) diets, or other structured therapeutic dietary protocols were excluded. Studies of single vitamin or mineral supplements, probiotics/prebiotics, or short-term experimental food challenges were also excluded. Case reports, case series, narrative reviews, systematic reviews, scoping reviews, study protocols, letters with no data, animal/in vitro studies, and conference abstracts that did not include sufficient methodological details were excluded. Studies were also excluded if data specific to the nutritional exposure of people with IBD were not reported separately, if no dietary exposure or relevant outcome was reported, or if the full article was not available in English.

Information Sources

In addition to the literature search in PubMed/MEDLINE, Scopus, and Web of Science Core Collection for literature from January 1, 2016, to August 4, 2026, the reference lists of relevant review articles and included studies were manually searched for additional potentially eligible papers. Search dates, information sources, and other reference-screening procedures were recorded to facilitate reporting and replication.

Search Strategy

A search strategy was formulated using keywords related to inflammatory bowel disease, dietary patterns, and dietary practices and/or controlled vocabulary terms, where applicable. The main search terms used were inflammatory bowel disease, lifestyle eating habits, and patient changes in eating habits. The Boolean operator “OR” was used between the search terms within each concept, and the Boolean operator “AND” was used between the main concepts. As appropriate, truncation, phrase searching, field tags, and database-specific indexing terms were used. Table 2 presents the key concepts and terms associated with each.

**Table 2:** Principal Concepts and Terms Used in the Literature Search

Search concept	Controlled vocabulary and free-text terms
Inflammatory bowel disease	“Inflammatory Bowel Diseases,” “inflammatory bowel disease,” IBD, “Crohn Disease,” “Crohn’s disease,” “Crohn disease,” “ulcerative colitis,” “Colitis, Ulcerative,” “indeterminate colitis,” “IBD-unclassified,” and IBD-U
Dietary patterns	“Dietary Patterns,” “dietary pattern,” “diet pattern,” “habitual diet,” “diet quality,” “dietary quality,” “dietary diversity,” “food frequency,” “Mediterranean diet,” “Western diet,” “plant-based diet,” “high-fat diet,” “high-sugar diet,” “high-fibre diet,” “low-fibre diet,” and “ultra-processed food”
Dietary practices	“dietary practice,” “dietary behaviour,” “dietary behavior,” “eating behaviour,” “eating behavior,” “food avoidance,” “food restriction,” “dietary restriction,” “restrictive eating,” “food elimination,” “elimination diet,” “trigger food,” “meal skipping,” fasting, “reduced food intake,” and “food choice”

The initial search strategy was designed in PubMed/MEDLINE and then modified to fit the search interface and indexing of both Scopus and Web of Science Core Collection. However, outcome-related terms were not included in the mandatory search parts of the query since search results may not mention gastrointestinal symptoms or outcomes of gastrointestinal disease. The searches were restricted to articles from January 1, 2016, to August 4, 2026. The searches were conducted on August 4, 2026, in PubMed/MEDLINE, Scopus, and Web of Science Core Collection. Full search strategies for each database are provided in the Appendices.

Study Selection

All records found in the database searches were imported into Zotero for study screening, duplicate detection, and reference management. Before screening, duplicate citations were detected using the duplicate detection function of the Zotero desktop app and were manually verified and merged. Titles and abstracts were independently examined by two reviewers and classified based on the predefined eligibility criteria. Records that appeared to be relevant were selected for full-text evaluation. Selected articles were then reviewed independently by the reviewers, and final inclusion was determined based on their assessments. Any disagreements during title/abstract screening or full-text assessment were resolved through discussion and consensus between the reviewers. If consensus could not be reached, a third reviewer was consulted to make the final decision. Reasons for exclusion at the full-text stage were systematically recorded. Multiple publications based on the same population were integrated and considered as one study, and complementary information was taken from the publications, if available. This complete selection process was documented using a PRISMA 2020 flow diagram.

Data Extraction

Data from the included studies were extracted using a data-extraction form created in Microsoft Excel (Microsoft Corporation, Redmond, Washington). The form was pilot-tested on a small number of the included studies and adjusted for consistency and completeness before full extraction. Two reviewers independently conducted the extraction, and discrepancies were resolved through discussion and consensus. The extracted data consisted of study identification, year of publication, country of study, study design, recruitment setting, sample size, participant characteristics, duration of follow-up (if available), IBD subtype, and disease activity status. Information on dietary exposure was also collected, including dietary pattern or practice, definition of exposure, method of dietary assessment, whether the instrument used for dietary assessment was validated, recall period of the dietary assessment, and whether the dietary assessment was self-directed or professionally advised. The outcome measures included gastrointestinal symptoms, clinical disease activity scores, inflammatory markers, endoscopy/histology, relapse, hospitalisation, treatment escalation, surgery, and nutritional/social outcomes, as applicable. Effect estimates, confidence intervals, p-values, and variables for multivariable analyses were obtained when reported. Any information that was missing or unclear was recorded as not reported.

Methodological Quality and Risk-of-Bias Assessment

The Joanna Briggs Institute (JBI) Critical Appraisal Tools 2020 [15] were used to analyse the methodological quality of the studies. Cross-sectional studies were assessed using the JBI Checklist for Analytical Cross-Sectional Studies, longitudinal cohort studies using the JBI Checklist for Cohort Studies, and case-control studies using the JBI Checklist for Case-Control Studies. The findings of the JBI critical appraisal were considered during the interpretation of the evidence, with methodological limitations and study quality informing the assessment of the consistency and reliability of reported associations. The appraisal domains were validity of dietary-exposure measurement, identification and control of confounding factors, validity of outcome assessment, completeness of follow-up (if applicable), appropriateness of participant recruitment, and clarity of inclusion criteria. Relevant clinically important potential confounders (such as disease activity, medication use, disease duration, smoking history, and previous surgery for IBD) were taken into account where relevant, and the potential for exposure misclassification and reverse causation was evaluated. Two independent reviewers conducted the quality appraisal, and any differences in opinion were addressed through discussion and consensus. The results did not include a numerical score but were presented for each methodological domain. No studies were omitted on the basis of appraisal findings, but methodological limitations were taken into account during the interpretation and synthesis of evidence.

Data Synthesis

Studies were synthesised using a structured narrative approach that emphasised study characteristics and findings. Study information was abstracted and summarised in evidence tables, including study design, population information, type of IBD, disease activity, dietary exposure, dietary assessment method, outcome measures, and main results. The synthesis was organised around the types of dietary exposures (global dietary pattern, high frequency of eating food or food groups, food avoidance, dietary restriction, meal pattern, and fasting). Gastrointestinal symptoms, clinical disease-activity indices, biochemical markers of inflammation, endoscopic and/or histological disease activity, relapse, hospitalisation, treatment escalation, surgery, and related nutritional and/or psychosocial consequences were all evaluated individually. Associations were reported as positive, inverse, absent, mixed, or unclear. The studies were interpreted taking into account study design, sample size, effect estimates, precision, adjustment for confounding, validity of exposure and outcome assessment, temporal relationship, and methodological quality. Cross-sectional dietary changes were interpreted with caution, as they could be a reaction to symptoms or active disease. A meta-analysis was not performed due to substantial heterogeneity in dietary exposures, assessment instruments, populations, disease activity measures, and reported outcomes. Therefore, findings were synthesised narratively using a structured approach.

Results

Study Selection

A total of 1,378 records were found in the database and supplementary searches in PubMed/MEDLINE, Scopus, and Web of Science Core Collection. Following the removal of 312 duplicate records, 1,066 unique records were left for title/abstract screening. Of these 1,066 records, 986 were excluded for not meeting the predefined eligibility criteria, and 80 reports were sought for retrieval. Of these, 15 reports could not be retrieved due to lack of full-text access. The remaining 65 articles were subsequently assessed for eligibility according to the predefined inclusion/exclusion criteria. Full-text evaluation led to the exclusion of 54 articles because they studied therapeutic dietary interventions, did not evaluate eligible habitual dietary patterns or patient-adopted dietary practices, did not include gastrointestinal symptoms or disease activity outcomes, used an inappropriate study design, or reported gastrointestinal data in insufficient detail to be separated from other data. The qualitative synthesis of this systematic review included the 11 articles that fulfilled all inclusion criteria. The studies analysed dietary patterns, food avoidance, dietary restriction, food-related behaviours, restrictive eating, gastrointestinal symptoms, clinical disease activity, inflammatory markers, and nutritional and/or psychosocial outcomes in patients with Crohn's disease, inflammatory bowel disease (IBD), and ulcerative colitis (UC). Studies were identified, screened, and assessed for eligibility following the process outlined in the PRISMA 2020 flow chart (Figure 1).

**Figure 1:**
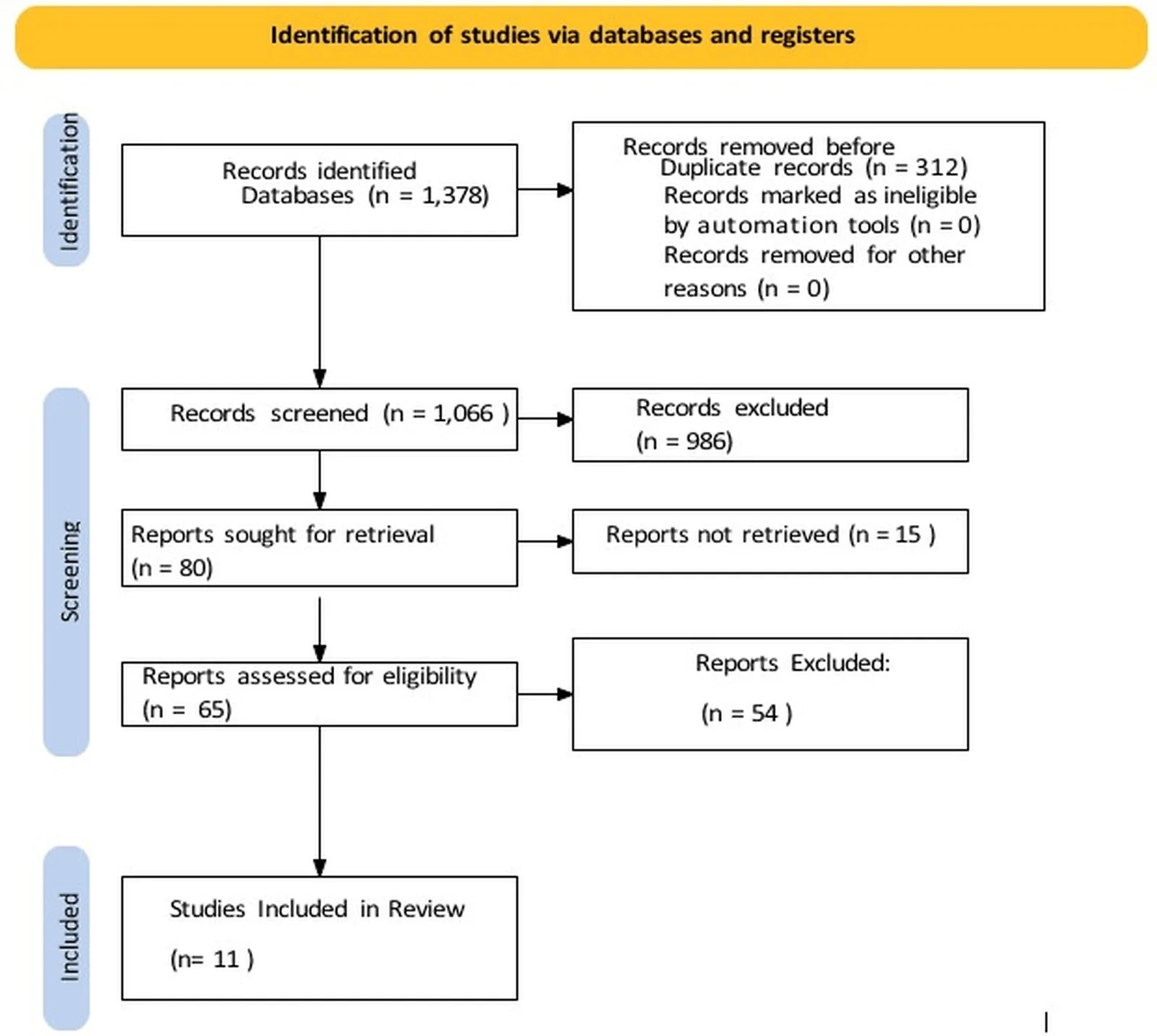
PRISMA 2020 flow diagram of study identification, screening, eligibility assessment, and inclusion PRISMA: Preferred Reporting Items for Systematic Reviews and Meta-Analyses.

Characteristics of the Included Studies

The characteristics of the included studies are summarised in Table 3. The studies were carried out in India, the Netherlands, Malaysia, New Zealand, the United Kingdom, the United States, China, Poland, and Australia, with publication dates ranging from 2017 to 2025. All the studies included here contributed data from 2,242 patients with IBD; the numbers of patients with IBD in individual studies ranged from 64 to 372. The available evidence was mostly observational and cross-sectional, with prospective cohort data with one-year follow-up from Koppelman et al. (2025) and baseline dietary information from a longitudinal cohort of patients with IBD from Cosier et al. (2024) analysed as a cross-sectional study [16-26].

**Table 3:** Characteristics and Principal Findings of the Included Studies aOR, adjusted odds ratio; ARFID, avoidant/restrictive food intake disorder; CD, Crohn’s disease; CDAI, Crohn’s Disease Activity Index; CI, confidence interval; CRP, C-reactive protein; DII, Dietary Inflammatory Index; FCP, faecal calprotectin; FFQ, food-frequency questionnaire; FrQoL, food-related quality of life; HBI, Harvey–Bradshaw Index; IBD, inflammatory bowel disease; IBD-U, inflammatory bowel disease-unclassified; NIAS, Nine-Item Avoidant/Restrictive Food Intake Disorder Screen; OR, odds ratio; PCA, principal component analysis; PG-SGA, Patient-Generated Subjective Global Assessment; PRO2, patient-reported outcome 2; SCCAI, Simple Clinical Colitis Activity Index; UC, ulcerative colitis. ARFID values refer to positive screening/risk rather than confirmed clinical diagnosis.

Study	Country	Study design	IBD sample	Population	Dietary exposure/assessment	Main outcomes and principal findings relevant to review
Tomar et al., 2017 [16]	India	Cross-sectional observational study	316	218 UC; 98 CD	Interview-administered questionnaire on dietary beliefs/practices, food avoidance, dietary preferences and intake, meal frequency, changes after diagnosis and during relapse	86% changed their diet after being diagnosed; 90% imposed restrictions. Modification and avoidance were higher during relapse than remission (92.65% vs 80.56%, p = 0.002; 94.85% vs 86.11%, p = 0.011). Approximately 40% were not eating as recommended; activity was measured using SCCAI/CDAI.
De Vries et al., 2019 [17]	Netherlands	Cross-sectional online questionnaire study	294	148 UC; 146 CD	37-item questionnaire evaluating nutrition knowledge, eating habits, food omissions, dietary guidance, and dietary attitudes	62% reported successful symptom control through dietary adaptation; 76.5% omitted foods. Common omissions included spicy/strongly seasoned foods, carbonated drinks, and dairy. The study could not distinguish symptom effects from effects on intestinal inflammation.
Shafiee et al., 2020 [18]	Malaysia	Cross-sectional study	64	UC only; 41 inactive, 23 active	Validated dietary-history assessment plus food-belief and food-avoidance questionnaires; Powell-Tuck Index	Food avoidance was more frequent in active than inactive UC (95.7% vs 43.9%); active UC had lower dietary intake. Both groups had inadequate intake of several macro- and micronutrients.
Morton et al., 2020 [19]	New Zealand	Cross-sectional retrospective questionnaire study	233	146 CD; 75 UC; 12 IBD-U	Self-administered questionnaire that measures 142 food, beverage, additive, and cooking habits	55% associated dietary components to symptom onset, 70% to exacerbation and 35% to symptom reduction. No significant difference was seen regarding the changes in type or proportion of elements involved in relation to the IBD subtype or recent disease activity.
Crooks et al., 2022 [20]	United Kingdom	Cross-sectional questionnaire-based observational study	208	Clinically inactive UC	27-item questionnaire on dietary practices, perceived triggers, food avoidance, exclusion diets, and information sources; PRO2 for clinical inactivity	59% actually avoided foods for fear of relapse even though their clinical history showed them to be clinically inactive. Female sex independently predicted food avoidance (OR 2.26, 95% CI 1.25–4.12; p = 0.007). Social effects were avoiding family meals (28%) and eating out (21%).
Robelin et al., 2021 [21]	United States	Single-centre cross-sectional pilot study	98	Adults with CD or UC	NIAS screening and clinician assessment of disease severity/recognition of eating disorder	10.2% screened positive for ARFID risk. Severe disease occurred in 40.0% of NIAS-positive vs 3.3% of NIAS-negative participants with severity data (p = 0.027). Clinician recognition sensitivity was 0% and specificity 96.5%.
Yelencich et al., 2022 [22]	United States	Cross-sectional ambulatory-clinic study	161	73 CD; 83 UC; 5 IBD-U	NIAS, food-avoidance assessment, PG-SGA; HBI/SCCAI; CRP, FCP and endoscopic assessment	17% screened positive for ARFID risk. Active symptoms (aOR 5.35, 95% CI 1.91–15.01) and objective inflammation (aOR 3.31, 95% CI 1.06–10.29) were independently associated with positive screening; malnutrition risk was 60.7% vs 15.8% (p < 0.01).
Yin et al., 2023 [23]	China	Multicentre cross-sectional study	372	Adults with CD or UC	Chinese NIAS and Food Literacy Evaluation Questionnaire; clinical activity measures	32.5% were identified as at risk for ARFID. Multivariable prediction was achieved by negative correlations with illness phenotype, disease activity, food planning/management, and food literacy (β = -0.299; p < 0.01) when assessed by NIAS.
Godala et al., 2023 [24]	Poland	Prospective questionnaire-based observational study (cross-sectional assessment)	82	48 CD; 34 UC	Questionnaire evaluating food restrictions, behaviours, and dietary views during remission and recurrence	85.4% believed diet could trigger relapse; approximately 75% modified diet after diagnosis and 81.7% imposed restrictions. CD patients more often than UC patients believed foods should be avoided to prevent relapse (91.7% vs 67.6%; p < 0.05).
Cosier et al., 2024 [25]	Australia	Cross-sectional analysis within a longitudinal IBD cohort	223 IBD	117 CD; 106 UC	Validated FFQ, 3-day food records, PCA/cluster analysis; fibre, polyphenol and fermented-food assessment; CDAI and FCP	Higher fibre intake was observed with no vs mild FCP inflammation (17.72 vs 14.62 g/day; overall p = 0.023). High CDAI adherence was associated with high CDAI adherence in CD (p = 0.004), whereas vegan style adherence was associated with FCP category in combined IBD (p = 0.015).
Koppelman et al., 2025 [26]	Netherlands	Prospective cohort study with one-year follow-up	191 at baseline	Adult IBD cohort; 53 active at baseline	Validated GINQ-FFQ; DII, Mediterranean Diet Score, Healthy Diet Indicator, ultra-processed-food intake and PCA; FrQoL; CRP/FCP activity	Higher FrQoL was associated with remission (p = 0.020). There was no significant association between diet quality/patterns and disease activity; and animal protein-rich pattern and convenience pattern had nonsignificant associations (OR 1.479, p = 0.088; OR 1.396, p = 0.060).

Most of the studies included patients with both Crohn's disease (CD) and ulcerative colitis (UC), but Crooks et al. (2021) and Shafiee et al. (2020) included patients with UC alone. Participants with an indeterminate diagnosis (IBD-unclassified, IBD-U) were included in the Morton et al. (2020) and Yelencich et al. (2022) cohorts. Study populations included patients with active disease, patients in remission, or those with mixed disease-activity states, and dietary behaviours and exposures were examined across disease activity states of IBD [18-20,22].

There was a wide range of approaches to dietary exposure assessment among the studies included. More recent studies used a variety of structured or self-administered questionnaires, such as food-frequency questionnaires, three-day food records, dietary-quality indices, principal component analysis, and the Nine-Item Avoidant/Restrictive Food Intake Disorder Screen (NIAS), to assess dietary beliefs, food avoidance, dietary restriction, changes in food intake after diagnosis or during relapse, meal-related behaviours, and self-imposed exclusion practices, respectively [18,21-23,25,26].

The heterogeneity of outcome assessment was also evident. In several studies, objective inflammatory indicators such as C-reactive protein (CRP), faecal calprotectin (FCP), and endoscopic evaluation were also included, allowing dietary exposures to be correlated with biochemical or endoscopic disease activity as well as symptom activity [22,25,26].

Methodological Quality of the Included Studies

The Joanna Briggs Institute (JBI) critical appraisal checklists were used to evaluate methodological quality using design-specific instruments [15]. A total of 10 studies were appraised with the JBI Checklist for Analytical Cross-Sectional Studies, and one study, Koppelman et al. (2025), was appraised with the JBI Checklist for Cohort Studies [16-26]. The detailed appraisal results for the analytical cross-sectional studies and the prospective cohort study are presented in Table 4 and Table 5, respectively. The JBI cross-sectional tool evaluates eight domains: sample selection, exposure and outcome measurement, identification and management of confounding, and statistical analysis; the cohort tool also examines comparability of exposure groups and completeness and adequacy of follow-up. Overall, the descriptions of the inclusion criteria and study settings were acceptable in most studies [16-26]. Studies that employed validated dietary instruments, NIAS screening, objective inflammatory markers, and standardised disease-activity indices had greater methodological strength [18,20-23,25,26]. Limitations occurred more often in questionnaire studies based on retrospective or self-reported dietary information [16,17,19,20,24]. Several studies made some adjustments for confounding, and Crooks et al. (2020), Yelencich et al. (2022), Yin et al. (2023), Cosier et al. (2024), and Koppelman et al. (2025) did so more explicitly [16,20,22,23,25,26]. Cross-sectional designs also restricted the ability of investigators to make inferences about temporal relationships and posed the risk of reverse causality. After methodological appraisal, no study was excluded.

**Table 4:** JBI Critical Appraisal of Analytical Cross-Sectional Studies JBI analytical cross-sectional domains: Q1, inclusion criteria clearly defined; Q2, subjects and setting described; Q3, exposure measured validly and reliably; Q4, objective standard criteria used to measure the condition; Q5, confounding factors identified; Q6, strategies used to address confounding; Q7, outcomes measured validly and reliably; Q8, appropriate statistical analysis. Y, yes; N, no; U, unclear. These represent the eight domains of the JBI Critical Appraisal Checklist for Analytical Cross-Sectional Studies.

Study	Q1	Q2	Q3	Q4	Q5	Q6	Q7	Q8
Tomar et al., 2017 [16]	Y	Y	U	Y	Y	N	Y	Y
De Vries et al., 2019 [17]	Y	Y	U	U	Y	N	U	Y
Shafiee et al., 2020 [18]	Y	Y	Y	Y	Y	N	Y	Y
Morton et al., 2020 [19]	Y	Y	U	Y	Y	N	U	Y
Crooks et al., 2021 [20]	Y	Y	U	Y	Y	Y	Y	Y
Robelin et al., 2021 [21]	Y	Y	Y	Y	N	N	Y	Y
Yelencich et al., 2022 [22]	Y	Y	Y	Y	Y	Y	Y	Y
Yin et al., 2023 [23]	Y	Y	Y	Y	Y	Y	Y	Y
Godala et al., 2023 [24]	Y	Y	U	Y	Y	N	U	Y
Cosier et al., 2024 [25]	Y	Y	Y	Y	Y	Y	Y	Y

**Table 5:** JBI Critical Appraisal of the Prospective Cohort Study JBI cohort domains: Q1, comparison groups similar and recruited from the same population; Q2, exposure measured similarly between groups; Q3, valid and reliable exposure measurement; Q4, confounding factors identified; Q5, strategies used to address confounding; Q6, participants free of the outcome at baseline; Q7, outcomes measured validly and reliably; Q8, sufficient follow-up duration; Q9, completeness of follow-up; Q10, strategies addressing incomplete follow-up; Q11, appropriate statistical analysis. Y, yes; N, no; U, unclear.

Study	Q1	Q2	Q3	Q4	Q5	Q6	Q7	Q8	Q9	Q10	Q11
Koppelman et al., 2025 [26]	Y	Y	Y	Y	Y	N	Y	Y	N	U	Y

Dietary Patterns and Diet Quality

Evidence specifically addressing habitual dietary patterns, nutrient adequacy, and overall diet quality was contributed by Shafiee et al. (2020), Cosier et al. (2024), and Koppelman et al. (2025) [18,25,26]. Shafiee et al. (2020) reported substantially more food avoidance in active than inactive ulcerative colitis (95.7% vs 43.9%) and lower dietary intake in active disease; both groups had inadequate intake of several nutrients, including protein, calcium, iron, folate, zinc, and vitamins D, B12, and E [18]. Cosier et al. (2024) found higher total dietary-fibre intake among participants without FCP-defined inflammation than among those with mild inflammation (17.72 vs 14.62 g/day; overall p = 0.023). In Crohn’s disease, CDAI differed across quartiles of adherence to a high plant-diversity pattern (p = 0.004), and in the combined IBD cohort, greater adherence to a vegan-style pattern was associated with a higher FCP category (p = 0.015); a meat-eater pattern was also associated with mild FCP-defined inflammation in Crohn’s disease [25]. Results from Koppelman et al. (2025) indicated overall poor diet quality and higher food-related quality of life in remission compared to active disease (p = 0.020), although no clear relationships between overall diet quality or dietary-pattern adherence and disease activity were found [26]. Overall, there was no consistent evidence of an association between higher diet quality and lower IBD activity, and there were mixed associations for individual dietary patterns by population and disease-activity measure.

Dietary Practices, Food Avoidance, and Restrictive Eating

Dietary modification and food avoidance were consistently reported across the included studies [16,17,19-24]. Tomar et al. (2017) found that approximately 86% of participants modified their diet after IBD diagnosis and 90% imposed food restrictions; dietary modification and food avoidance were both more frequent during relapse than remission (92.65% vs 80.56%, p = 0.002; and 94.85% vs 86.11%, p = 0.011, respectively) [16]. According to De Vries et al. (2019), almost 77% of participants avoided certain items, most often spicy or highly seasoned meals, carbonated drinks, dairy products, energy drinks, deep-fried foods, and alcohol; 62% of respondents thought dietary modification was effective in controlling symptoms [17]. Crooks et al. (2020) observed that 59% of patients with clinically inactive ulcerative colitis avoided foods to prevent relapse; female sex independently predicted food avoidance (OR 2.26, 95% CI 1.25-4.12; p = 0.007) [20]. Godala et al. (2023) found that 85.4% believed that their diet could trigger a relapse, around 75% changed their eating habits after diagnosis, and 81.7% applied dietary restrictions to prevent relapse [24]. Similarly, in studies focusing on ARFID, Robelin et al. (2021) found positive NIAS screening in 10.2% of their sample, Yelencich et al. (2022) in 17%, and Yin et al. (2023) in 32.5% [21-23]. In general, there was a high prevalence of restrictive dieting during both active disease and remission, and this was mostly related to symptom avoidance and risk of relapse.

Associations Between Diet and Gastrointestinal Symptoms

Several included studies demonstrated strong patient-perceived associations between dietary intake and gastrointestinal symptoms [16,17,19,22,24]. Morton et al. (2020) reported that 55% of participants associated one or more dietary elements with symptom onset, 70% with symptom exacerbation, and 35% with symptom reduction; importantly, the type or number of implicated dietary elements did not differ significantly by IBD subtype or recent disease activity [19]. De Vries et al. (2019) reported that 62% found dietary adaptation to be successful in controlling symptoms and that food omission was more prevalent with spicy and strongly seasoned foods, carbonated drinks, and dairy products [17]. In general, there was a decrease in the consumption of various foods, although certain foods were perceived as being beneficial during relapses (77%, according to Tomar et al. (2017)) [16]. Similarly, during relapse, there was a high prevalence of symptom-driven dietary changes and food exclusion, as reported by Godala et al. (2023) [24]. According to Yelencich et al. (2022), 92% of participants with active symptoms and 74% of those with inactive symptoms avoided at least one food [22]. In general, symptom-related dietary modifications were commonly reported, but the cross-sectional nature of the evidence did not allow for determination of whether dietary exposures occurred before symptoms changed.

Associations With Disease Activity and Inflammation

Associations between dietary exposures and disease activity were inconsistent across the included studies [16,18,20-23,25,26]. Tomar et al. (2017) showed that dietary modification and food avoidance were more frequent during relapse than remission [16]. Shafiee et al. (2020) found that, according to the Powell-Tuck Index, patients with active UC had higher levels of food avoidance and dietary intake than patients with inactive UC [18]. Yelencich et al. (2022) found that active gastrointestinal symptoms (adjusted OR 5.35, 95% CI 1.91-15.01) and objective inflammation (adjusted OR 3.31, 95% CI 1.06-10.29) were independently associated with a positive ARFID-risk screen, with inflammation defined by either CRP, FCP, or endoscopic activity [22]. Yin et al. (2023) likewise identified disease phenotype, disease activity, and food-literacy dimensions as predictors of NIAS scores [23]. In Crohn's disease, Cosier et al. (2024) found that the high plant-diversity pattern was associated with CDAI (p = 0.004); in the combined IBD cohort, the vegan-style pattern was associated with FCP category (p = 0.015) [25]. Koppelman et al. (2025) did not report a significant association between overall diet quality and disease activity, although there was a nonsignificant trend toward more disease activity with an animal protein-rich diet (OR 1.479, 95% CI 0.943 to 2.319; p = 0.088) [26]. In sum, associations with disease activity were inconsistent and varied by dietary exposure and outcome measure. The principal associations between dietary exposures and gastrointestinal, inflammatory, nutritional, and behavioural outcomes across the included studies are summarised in Table 6.

**Table 6:** Summary of Associations Between Dietary Exposures and Outcomes ARFID, avoidant/restrictive food intake disorder; CD, Crohn's disease; CI, confidence interval; FCP, faecal calprotectin; NIAS, Nine-Item Avoidant/Restrictive Food Intake Disorder Screen; OR, odds ratio; UC, ulcerative colitis. Where a specific p-value or 95% CI was not reported by the original study, this is stated explicitly.

Study	Exposure	Outcome	Effect estimate/finding	95% CI or p-value	Adjustment	Interpretation
Tomar et al., 2017 [16]	Dietary modification and food avoidance	Relapse versus remission	Dietary modification: 92.65% during relapse vs 80.56% during remission. Food avoidance: 94.85% in relapse; 86.11% in remission.	Dietary modification: p = 0.002; food avoidance: p = 0.011. 95% CI not reported.	Relapse/remission comparisons at this site were made without making any multivariable adjustment.	Dietary modification and avoidance were significantly more frequent during relapse; the cross-sectional design does not establish directionality.
De Vries et al., 2019 [17]	Dietary adaptation and food omission	Patient-reported symptom control/food omission	62% reported successful symptom control through dietary adaptation; 76.5% omitted foods to reduce disease symptoms.	No p-value or 95% CI reported for these specific descriptive proportions.	Descriptive and bivariate analyses; no multivariable adjustment reported.	The cross-sectional design does not allow for inferences about directionality; dietary changes/avoidance were significantly higher during relapse.
Shafiee et al., 2020 [18]	Food avoidance, dietary intake and dietary beliefs	Active versus inactive UC	Food avoidance: 95.7% active vs 43.9% inactive UC. Active UC also had lower intake of several nutrients.	Food avoidance: p < 0.05 (exact p not reported). Diet-important belief: p = 0.001. Several nutrient comparisons were also significant; 95% CIs not reported.	No multivariable adjustment was highlighted.	Active UC was associated with greater food avoidance and poorer dietary intake; reverse causality remains possible.
Morton et al., 2020 [19]	Foods, beverages, additives and cooking methods	Symptom onset, exacerbation and reduction	55% linked dietary elements with symptom onset, 70% with exacerbation and 35% with symptom reduction; no significant differences were reported by IBD subtype or recent disease activity.	Headline proportions were descriptive; selected CD-vs-UC comparisons: onset p = 0.330; exacerbation p = 0.429.	Unadjusted comparisons; no multivariable adjustment reported.	Patient-perceived diet-symptom associations were noted but not consistently associated with IBD subtype or recent disease activity.
Crooks et al., 2021 [20]	Food avoidance in clinically inactive UC	Predictors of food avoidance to prevent relapse	59% avoided foods to prevent relapse; female sex independently predicted dietary avoidance.	Adjusted OR 2.26, 95% CI 1.25-4.12; p = 0.007.	Multifactorial binary logistic regression.	Food avoidance persisted despite clinical remission and was associated with factors beyond overt inflammatory activity.
Robelin et al., 2021 [21]	Positive NIAS screen/ARFID risk	Clinician-rated IBD severity	10.2% screened positive for ARFID risk. Severe disease occurred in 40.0% of NIAS-positive vs 3.3% of NIAS-negative participants with severity data.	p = 0.027; 95% CI not reported.	Unadjusted group comparison; no adjusted effect estimate reported.	ARFID risk was associated with greater disease severity, although the small pilot sample limits inference.
Yelencich et al., 2022 [22]	Positive ARFID-risk screen/restrictive eating	Active symptoms, objective inflammation and malnutrition risk	Active symptoms and objective inflammation were independently associated with ARFID-risk screening; malnutrition risk was 60.7% in ARFID-positive vs 15.8% in ARFID-negative participants.	Active symptoms: OR 5.35, 95% CI 1.91-15.01; objective inflammation: OR 3.31, 95% CI 1.06-10.29; malnutrition comparison: p < 0.01.	Multivariable logistic regression for clinical predictors, symptoms and inflammation modelled separately due to collinearity.	Objective inflammation and symptom burden also predicted restrictive-eating risk, which was linked to substantial nutritional risk.
Yin et al., 2023 [23]	ARFID risk/NIAS score, disease characteristics and food planning/management	NIAS score/restrictive-eating risk	In stepwise regression, UC phenotype, food planning/management and remission were negative predictors of NIAS score.	Food planning/management: beta = -0.299 in Model 2, p < 0.01; final Model 3: UC beta = -0.298, food planning/management beta = -0.279, remission beta = -0.275; all p < 0.01. 95% CIs were not reported.	Stepwise multivariable linear regression; final model R^2^ = 0.315, adjusted R^2^ = 0.310.	Higher NIAS scores were associated with Crohn disease phenotype, active disease and lower food planning/management scores; these are screening-risk associations, not proof of causation.
Godala et al., 2023 [24]	Dietary beliefs, modification and food restriction	Dietary behaviour/beliefs by IBD subtype and relapse/remission	75.6% modified diet after diagnosis and 81.7% imposed restrictions to prevent relapse. CD patients more often than UC patients believed foods should be avoided to prevent relapse (91.7% vs 67.6%).	p < 0.05; exact p value and 95% CI not reported.	Descriptive statistics and chi-square testing; no multivariable adjustment reported.	Dietary restriction was common and differed by IBD subtype for some beliefs, but causal effects cannot be established.
Cosier et al., 2024 [25]	Habitual dietary patterns and fibre intake	CDAI and FCP-defined inflammation	Total fibre was different between the FCP groups among 223 IBD participants (117 CD and 106 UC); CDAI was different between the high adherence quartiles of plant diversity for CD, and vegan-style adherence was associated with FCP category in combined IBD.	Total fibre across FCP groups: p = 0.023, no-vs-mild: p = 0.025, insoluble fibre: p = 0.011, soluble fibre: p = 0.043, high plant-diversity/CDAI: p = 0.004, vegan-style/FCP: p = 0.015.	Cross-sectional dietary-pattern and group-comparison analysis and logistic binomial regression for PCA associations of significance.	Specific dietary patterns and fibre intake were statistically correlated with clinical or biochemical activity, but could not be established to be causal and temporal.
Koppelman et al., 2025 [26]	Overall diet quality and PCA-derived dietary patterns	Change in biochemical disease activity over one year	Overall diet quality was not associated, and animal protein-rich and convenience patterns were not significantly associated over time.	Animal protein-rich: OR 1.479, 95% CI 0.943-2.319, p = 0.088; convenience: OR 1.396, 95% CI 0.986-1.976, p = 0.060.	Multinomial logistic regression.	No diet quality consistently correlated with inflammatory activity, and the pattern associations were not statistically significant.

Avoidant/Restrictive Food Intake Disorder Risk

Avoidant/restrictive food intake disorder (ARFID) was specifically assessed in three studies that were included [21-23]. The results were consistent with the findings of Robelin et al. (2021), who found that 40.0% of those with data related to clinical severity were severe when they were also NIAS positive (3.3% when they were NIAS negative, p = 0.027), with a sensitivity of 0% and a specificity of 96.5% for the clinician diagnosis of an eating disorder. In regard to active gastrointestinal symptoms, Yelencich et al. (2022) found that gastrointestinal symptoms were an independent predictor of positive ARFID-risk screening (adjusted OR 5.35, 95% CI 1.91-15.01) and objective inflammation was an independent predictor of positive ARFID-risk screening (adjusted OR 3.31, 95% CI 1.06-10.29). For those who were positive, there was also a significant association with a high risk of malnutrition (60.7% vs 15.8%, p < 0.01) [22]. Yin et al. (2023) reported the highest prevalence, with 32.5% screening as high risk for ARFID. In multivariable modelling, the inverse association between NIAS scores and food literacy was maintained, as were disease phenotype, disease activity, and food-planning/management [23]. These studies relied on screening tools, not clinical diagnosis, and so reflect risk for ARFID, not a clinical diagnosis of ARFID. Overall, there was a relatively high proportion who screened positive for ARFID, and the risk of ARFID was linked to active symptoms, measures of disease activity or inflammation, lower food literacy, and nutritional vulnerability.

Nutritional and Psychosocial Consequences

Several studies included revealed nutritional and psychosocial impacts of dietary limitations in IBD [16,18,20-24,26]. Tomar et al. (2017) reported that about 40% of participants were not consuming enough food, with the amount further reduced during relapse, suggesting potential undernutrition [16]. Shafiee et al. (2020) found inadequate intake of multiple macro- and micronutrients in both active and inactive ulcerative colitis, with poorer intake and more frequent food avoidance among patients with active disease [18]. Yelencich et al. (2022) demonstrated that participants screening positive for ARFID were considerably more likely to be at risk of malnutrition than those screening negative (60.7% vs 15.8%; p < 0.01) [22]. Dietary restriction also affected daily and social eating behaviours. Crooks et al. reported that 28% avoided the same meal as their family and 21% avoided eating out at least sometimes; recent relapse independently predicted avoidance of the family meal (OR 2.60, 95% CI 1.24-5.46; p = 0.01), while female sex predicted avoidance of eating out (OR 2.84, 95% CI 1.41-5.75; p = 0.004) [20]. Additionally, Koppelman et al. (2025) found that food-related quality of life was higher in remission than in active disease (p = 0.020) [26]. In general, the association of restrictive eating with nutritional inadequacy, risk of nutritional inadequacy, decreased appetite, and negative food-related social and quality-of-life outcomes supported these findings.

Overall Synthesis of Findings

In the 11 studies included, dietary modification, food avoidance, and restrictive eating were common among patients with IBD, especially during symptomatic or active disease [16-24]. Commonly reported practices involved restricting consumption of perceived trigger foods, modifying their normal eating habits, and decreasing food consumption to help manage symptoms or prevent relapse [16,17,20,24]. Associations with gastrointestinal symptoms were more consistent than those with objective inflammatory measures; results from CRP levels, faecal calprotectin, endoscopic assessment, and validated disease-activity indices were more diffuse [18,22,23,25,26]. Habitual dietary-pattern studies found no single pattern linked to reduced disease activity, with some plant-based diet patterns and none of the animal-based diet patterns being associated with clinical or biochemical outcomes [25,26]. Studies that focused on ARFID achieved positive screening results of 10.2% to 32.5% of participants, higher scores with active symptoms, inflammation or disease activity, lower food literacy, and malnutrition [21-23]. Nutritional and psychosocial outcomes were poor nutrient intake, undernutrition/malnutrition risk, and food-related social functioning and quality of life [16,18,20,22,24,26]. Overall, the evidence suggests that dietary behaviours in IBD are strongly symptom-responsive, whereas their relationship with underlying inflammatory activity remains less consistent.

Discussion

This systematic review identified consistent patterns of food avoidance and restrictive eating among patients with IBD, while associations between dietary patterns and objective inflammatory activity remained inconsistent. Food avoidance, dietary changes, and restrictive eating were the most consistent behaviours identified across populations and disease states. These dietary practices were more consistently associated with gastrointestinal symptom burden, whereas associations with objective inflammatory activity were heterogeneous and insufficient to establish a consistent diet-inflammation relationship. Dietary practices were more consistently associated with gastrointestinal symptoms than with objective inflammatory activity, and several studies have shown associations with disease-activity indices, CRP, or faecal calprotectin, though not consistently [18,22,23,25,26]. Current treat-to-target guidelines also show that biochemical and endoscopic control of inflammation should be separated from symptomatic response [13].

Spicy foods, fatty foods, dairy, alcohol, coffee, high-fibre foods, and other perceived triggers were repeatedly avoided [16,17,20,24]. The association between food consumption and gastrointestinal symptoms should, however, be treated with caution. Foods may not exacerbate intestinal inflammation, but they can worsen abdominal discomfort, diarrhoea, bloating, urgency, or changes in bowel movements. This makes it more likely that diet-symptom relationships are more robust and consistent than relationships with inflammatory markers. The distinction is also consistent with the current clinical understanding that there are some dietary changes associated with inflammatory disease control, but not necessarily symptom management in patients with IBD who have continued gastrointestinal symptoms.

Another explanation for the results is reverse causation. The majority of the studies used in this review were cross-sectional, where dietary behaviours and outcomes were measured at a similar time. As the disease progresses, patients may gradually avoid food, cut down on how much they eat, or simply stop eating due to the severity of their symptoms. If there is an association between restriction and increased symptom burden, it is impossible to determine if restriction caused or preceded symptoms. This methodological limitation was specifically present in studies based on retrospective reporting of food triggers or dietary behaviours [16,17,19,20,24]. Based on the present results, it seems prudent to take a cautious approach in interpreting patient-perceived relationships between food and disease and to underscore the importance of prospective studies where dietary exposures are assessed prior to the onset of symptom change and inflammatory activity.

The evidence on overall dietary patterns and diet quality was inconsistent. Cosier et al. (2024) found associations between selected dietary patterns and clinical activity or faecal calprotectin, while Koppelman et al. (2025) did not establish any definitive link between the overall diet quality score or dietary-pattern adherence score and biochemical disease activity [25,26]. A recent systematic review of observational studies also highlighted the inconsistent findings regarding the association between diet-quality indices and inflammatory markers, disease activity, and quality of life in patients with IBD, with some suggesting more favourable associations with healthier or less inflammatory dietary patterns [27].

The findings also indicate that restrictive eating behaviours may have important clinical implications. The clinical relevance of this problem is further supported by additional recent evidence in which positive ARFID-risk screening was linked to active symptoms, disease severity, lower food literacy, and malnutrition risk [21-23]. In patients with quiescent IBD and IBS-like symptoms, a cross-sectional study in 2026 emphasised the need to identify restrictive eating behaviours even in the absence of inflammatory symptoms [28].

The nutritional information is in line with the current dietary recommendations. The updated ESPEN guideline emphasises the importance of nutritional assessment in IBD and recognises malnutrition as a major clinical concern requiring individualised management [11]. Similarly, the British Dietetic Association consensus guideline highlights both the integral role of nutrition in IBD management and the limitations of high-quality evidence supporting many dietary approaches [29]. These recommendations support the present review's finding that extensive self-directed restriction may be problematic when it compromises dietary diversity, nutrient intake, appetite, or food-related quality of life [10,18,22,24,26].

A few clinical implications are drawn next. Dietary counselling should differentiate between foods associated with gastrointestinal symptom triggers and dietary exposures that may influence underlying inflammatory activity. Any patient who is reporting significant dietary restrictions, weight loss, not eating, skipping meals, fear of eating, or avoiding a variety of food groups should be assessed for nutritional status and, if appropriate, referred to an IBD-experienced dietitian. Objective evaluation of inflammatory activity is also important when persistent symptoms are attributed to diet, because symptom burden alone may not indicate active mucosal inflammation [13]. Current ESPEN guidance supports individualised rather than indiscriminate dietary management in IBD [11].

This review has several strengths, such as the PECO criteria, a pre-designed search strategy, a structured search in the three major bibliographic databases, the homogeneity of the data extracted, and the separation of habitual dietary patterns from those adopted by patients and gastrointestinal symptoms from objective inflammatory outcomes. However, cross-sectional studies predominated in the evidence base, dietary assessment was often self-reported, and confounding was not always taken into account. The JBI critical appraisal findings were considered when interpreting the evidence, particularly when assessing the reliability and consistency of associations reported by the included studies. Studies with methodological limitations were not excluded but were considered when drawing conclusions regarding dietary practices, gastrointestinal symptoms, and inflammatory outcomes. There was significant variation among dietary definitions, exposure instruments, populations, and measures of disease activity. Due to substantial heterogeneity in dietary exposures, assessment instruments, disease-activity measures, and reported effect estimates, a meta-analysis was not performed. Another potential limitation is language and publication bias, which may be present due to restriction to English-language, peer-reviewed publications.

Future studies should include sufficiently sized prospective cohorts with repeated validated dietary questionnaires and contemporaneous CRP, faecal calprotectin, endoscopic activity, and patient-reported symptom measures. Common terms for food avoidance, restrictive eating, and ARFID risk should also be used. These studies would help clarify whether dietary changes precede inflammation or if they are mostly behavioural reactions to inflammation.

## Conclusions

This systematic review confirms that dietary modification, food avoidance, and restrictive eating habits are consistently reported among patients with IBD and are strongly linked to symptoms of gastrointestinal involvement rather than measures of inflammatory activity. Habitual dietary patterns or overall diet quality were inconsistently associated with clinical, biochemical, or endoscopic disease activity in the studies included, and no single dietary pattern proved conclusively beneficial or harmful. These behaviours may have clinically relevant implications, including nutritional compromise and reduced food-related quality of life. The results underscore the importance of differentiating symptom-provoking foods from dietary exposures that may affect bowel inflammation and of avoiding unnecessary exclusion of large food groups without appropriate dietary assessment and clinical evaluation. Dietary management should therefore be individualised and incorporated into the multidisciplinary management of IBD, and nutritional assessment should be undertaken, with access to dietetic support from someone experienced in managing nutritional issues for those with restrictive eating or nutritional compromise. Further prospective research with validated dietary tools and standardised clinical, biochemical, endoscopic, and/or patient outcomes is needed to establish whether certain dietary behaviours are risk factors for, or protective against, inflammatory disease activity.

## Appendices

**Table 7:** Database-specific search strategies

Database	Search Date	Search strategy
PubMed/MEDLINE	4 August 2026	("Inflammatory Bowel Diseases"[Mesh] OR "inflammatory bowel disease"[Title/Abstract] OR IBD[Title/Abstract] OR "Crohn Disease"[Mesh] OR "Crohn disease"[Title/Abstract] OR "Crohn's disease"[Title/Abstract] OR "Colitis, Ulcerative"[Mesh] OR "ulcerative colitis"[Title/Abstract] OR "indeterminate colitis"[Title/Abstract] OR "IBD-unclassified"[Title/Abstract] OR IBD-U[Title/Abstract]) AND ("Dietary Patterns"[Mesh] OR "dietary pattern*"[Title/Abstract] OR "diet pattern*"[Title/Abstract] OR "habitual diet*"[Title/Abstract] OR "diet quality"[Title/Abstract] OR "dietary quality"[Title/Abstract] OR "dietary diversity"[Title/Abstract] OR "food frequency"[Title/Abstract] OR "Mediterranean diet"[Title/Abstract] OR "Western diet"[Title/Abstract] OR "plant-based diet"[Title/Abstract] OR "ultra-processed food*"[Title/Abstract] OR "dietary practice*"[Title/Abstract] OR "dietary behavior*"[Title/Abstract] OR "dietary behavior*"[Title/Abstract] OR "eating behavior*"[Title/Abstract] OR "eating behavior*"[Title/Abstract] OR "food avoidance"[Title/Abstract] OR "food restriction"[Title/Abstract] OR "dietary restriction"[Title/Abstract] OR "restrictive eating"[Title/Abstract] OR "food elimination"[Title/Abstract] OR "elimination diet*"[Title/Abstract] OR "trigger food*"[Title/Abstract] OR "meal skipping"[Title/Abstract] OR fasting[Title/Abstract] OR "reduced food intake"[Title/Abstract] OR "food choice*"[Title/Abstract])
Scopus	4 August 2026	TITLE-ABS-KEY("inflammatory bowel disease" OR IBD OR "Crohn disease" OR "Crohn's disease" OR "ulcerative colitis" OR "indeterminate colitis" OR "IBD-unclassified" OR IBD-U) AND TITLE-ABS-KEY("dietary pattern*" OR "diet pattern*" OR "habitual diet*" OR "diet quality" OR "dietary quality" OR "dietary diversity" OR "food frequency" OR "Mediterranean diet" OR "Western diet" OR "plant-based diet" OR "ultra-processed food*" OR "dietary practice*" OR "dietary behavior*" OR "eating behavior*" OR "food avoidance" OR "food restriction" OR "dietary restriction" OR "restrictive eating" OR "food elimination" OR "elimination diet*" OR "trigger food*" OR "meal skipping" OR fasting OR "reduced food intake" OR "food choice*")
Web of Science Core Collection	4 August 2026	TS=("inflammatory bowel disease" OR IBD OR "Crohn disease" OR "Crohn's disease" OR "ulcerative colitis" OR "indeterminate colitis" OR "IBD-unclassified" OR IBD-U) AND TS=("dietary pattern*" OR "diet pattern*" OR "habitual diet*" OR "diet quality" OR "dietary quality" OR "dietary diversity" OR "food frequency" OR "Mediterranean diet" OR "Western diet" OR "plant-based diet" OR "ultra-processed food*" OR "dietary practice*" OR "dietary behavior*" OR "eating behavior*" OR "food avoidance" OR "food restriction" OR "dietary restriction" OR "restrictive eating" OR "food elimination" OR "elimination diet*" OR "trigger food*" OR "meal skipping" OR fasting OR "reduced food intake" OR "food choice*")
